# Supplementary material for: Lipid-Based Nanocarriers for Delivery of Neuroprotective Kynurenic Acid: Preparation, Characterization, and BBB Transport
Source: Int J Mol Sci. 2023 Sep 18;24(18):14251. doi: 10.3390/ijms241814251 (PMC10531491; doi:10.3390/ijms241814251)
Supplement: Supplementary file 1 [file ijms-24-14251-s001.zip › ijms-2604494-supplementary.pdf]

International Journal of *Molecular Sciences*

MDPI

*Supplementary Materials*

# Lipid-Based Nanocarriers for Delivery of Neuroprotective Kynurenic Acid: Preparation, Characterization, and BBB Transport

**Ádám Juhász [1,2,*], Ditta Ungor [2], Norbert Varga [1,2], Gábor Katona [3], György T. Balogh [4,5] and Edit Csapó [1,2,*]**

1 Interdisciplinary Excellence Center, Department of Physical Chemistry and Materials Science, University of Szeged, Rerrich B. Sqr. 1, H-6720 Szeged, Hungary; vargano@chem.u-szeged.hu
2 MTA-SZTE Lendület "Momentum" Noble Metal Nanostructures Research Group, University of Szeged, Rerrich B. Sqr. 1, H-6720 Szeged, Hungary; ungord@chem.u-szeged.hu
3 Institute of Pharmaceutical Technology and Regulatory Affairs, Faculty of Pharmacy, University of Szeged, Eötvös Str. 6, H-6720 Szeged, Hungary; katona.gabor@szte.hu
4 Department of Pharmaceutical Chemistry, Semmelweis University, Hőgyes Endre út 9., H-1092 Budapest, Hungary; balogh.gyorgy@vbk.bme.hu
5 Department of Chemical and Environmental Process Engineering, Budapest University of Technology and Economics, Műegyetem Rakpart 3, H-1111 Budapest, Hungary
* Correspondence: juhaszad@chem.u-szeged.hu (Á.J.); juhaszne@chem.u-szeged.hu (E.C.)

## 1. Optimization of the preparation of the liposomal particles

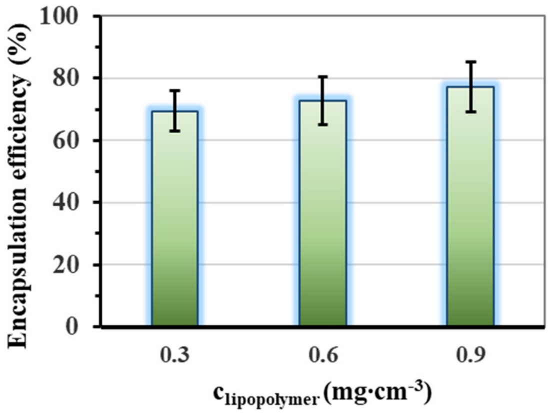


**Figure S1.:** Change of the encapsulation efficiency of the WSLP carriers as a function of the applied lipopolymer concentration ($c_{KYNA}$ = 1.0 mM)

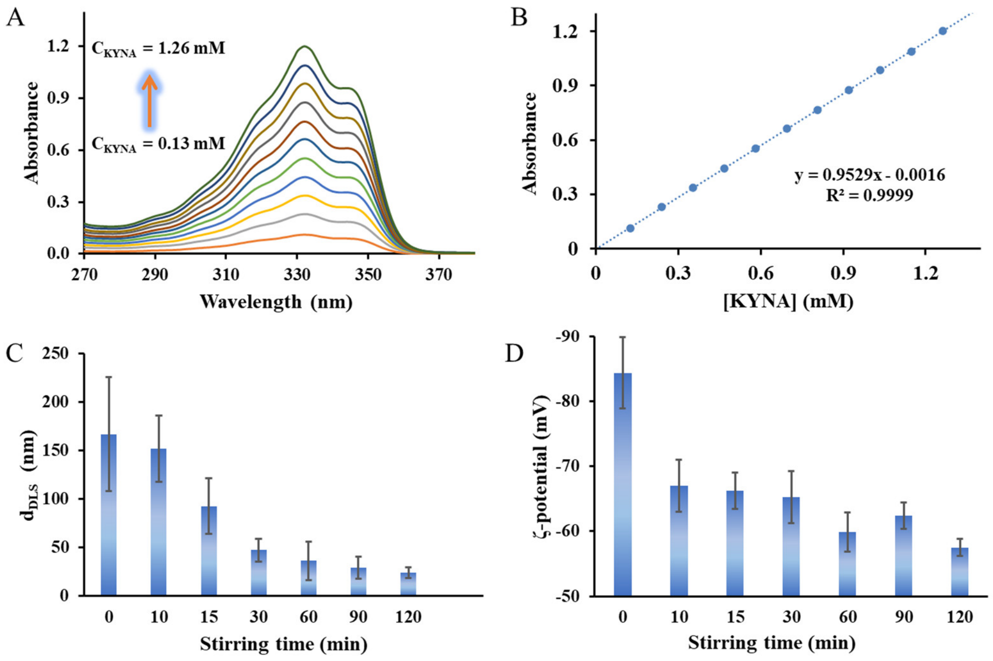


**Figure S2.: A:** Uv-VIS spectrum series of aqueous KYNA solutions for determination of the relationship between the measured absorbance (at 332 nm) and the applied drug concentration. **B:** Calibration curve for determination of the encapsulation efficiency (EE%) of liposomal carriers. **C:** Modifying of the hydrodynamic diameter (nm) of the lipid-based carriers as a function of the applied stirring time. **D:** Altering of the ζ-potential (mV) of the lipid-based carriers as a function of the applied stirring time.

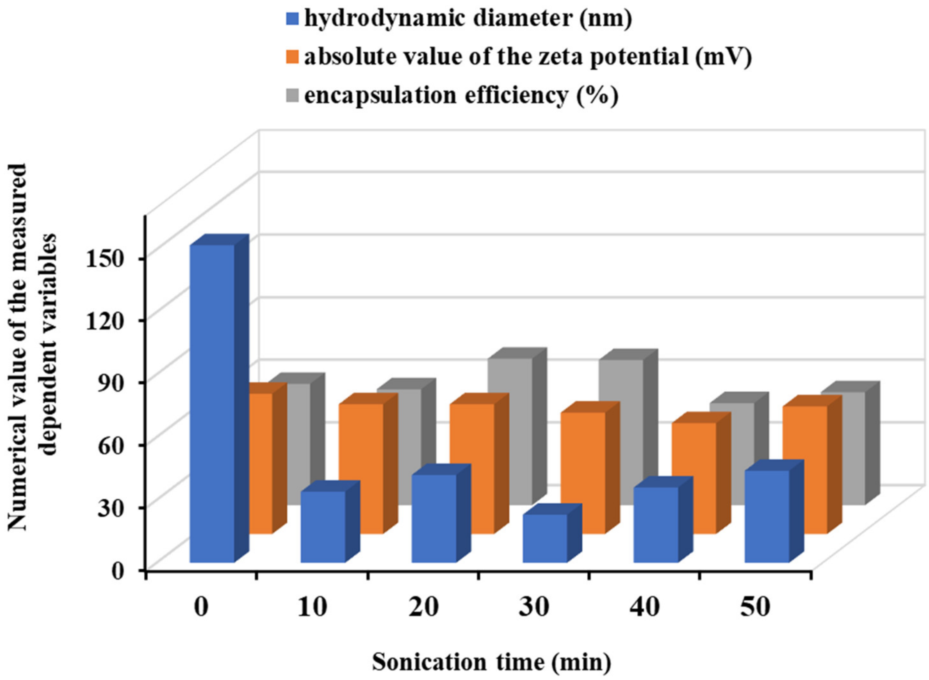


**Figure S3.:** Alternation of the measured dependent properties (hydrodynamic diameter / nm; absolute value of the zeta potential / mV and encapsulation efficiency / %) of the LIP carriers against the time of sonication.

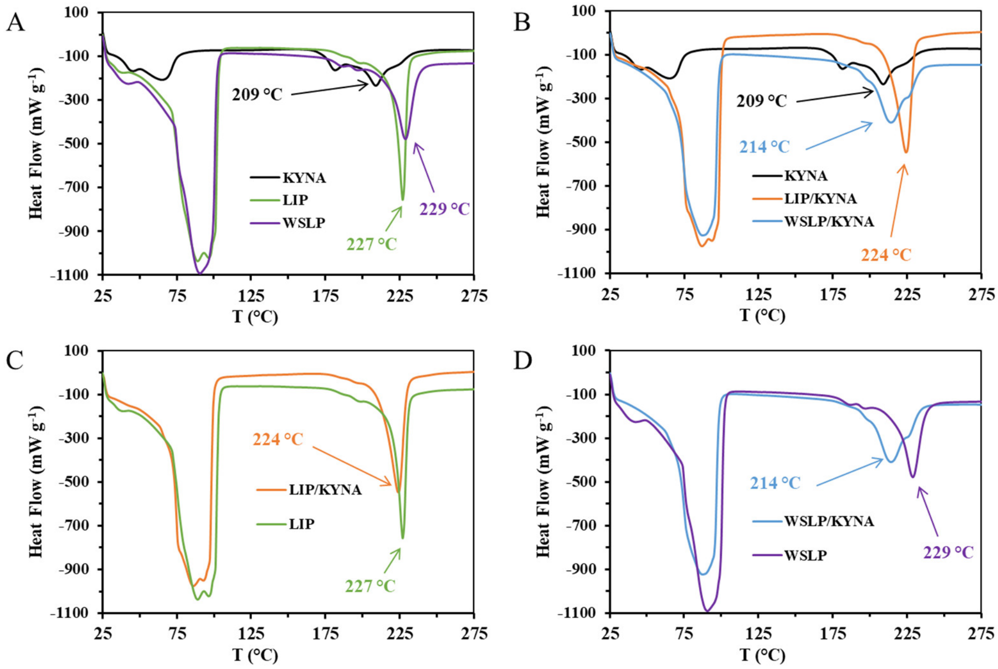


**Figure S4.:** Differential scanning calorimetry (DSC) curves of pure drug (KYNA as black line), asolectin based liposomes (LIP as green line), water soluble lipopolymer based liposomes (WSLP as lilac curve) and drug loaded liposomal carriers (LIP/KYNA carrier as orange line and WSLP/KYNA carrier as blue line) presented in different comparisons.

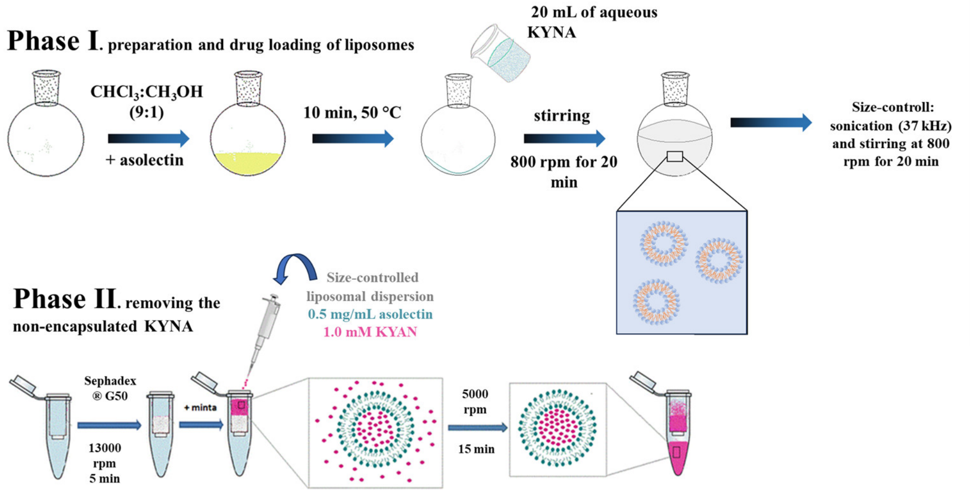


**Figure S5.:** Schematic representation of the preparation pathway of asolectin-based nanocarriers containing KYNA.

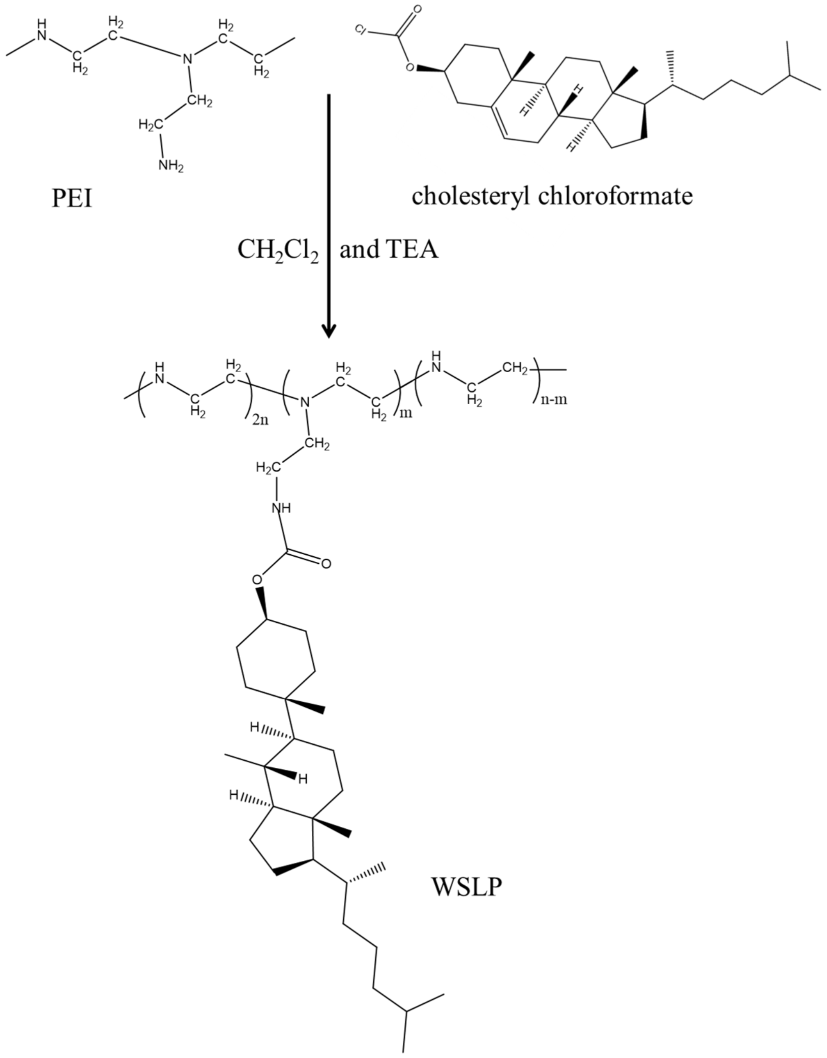


**Figure S6.:** Synthesis scheme of poly{(ethylenimine)-co-[N-2-(aminoethyl)ethylenimine]-co-[N-(N-cholesteryloxycarbonyl-(2-aminoethyl))ethylenimine]}.

**Table S1.** Transport of the free drug and liposomes (LIP and WSLP) containing KYNA through the in vitro human BBB model.

| **sample** | **Pe · $10^{-6}$ (min)** | **MR%** | **Flux · $10^{-6}$ ()** |
|---|---|---|---|
| KYNA | 4.0 ± 0.35 | 0.45 ± 0.36 | 1.55 ± 0.13 |
| LIP/KYNA | 21.3 ± 0.83 | 62.3 ± 0.5 | 5.29 ± 0.21 |
| WSLP/KYNA | 11.9 ± 0.45 | 23.7 ± 1.5 | 4.75 ± 0.18 |
